# Two common functional catalase gene polymorphisms (rs1001179 and rs794316) and cancer susceptibility: evidence from 14,942 cancer cases and 43,285 controls

**DOI:** 10.18632/oncotarget.10617

**Published:** 2016-07-15

**Authors:** Kang Liu, Xinghan Liu, Meng Wang, Xijing Wang, Huafeng Kang, Shuai Lin, Pengtao Yang, Cong Dai, Peng Xu, Shanli Li, Zhijun Dai

**Affiliations:** ^1^ Department of Oncology, Second Affiliated Hospital of Xi'an Jiaotong University, Xi'an, China

**Keywords:** catalase, polymorphism, cancer, susceptibility, meta-analysis

## Abstract

Recent studies have focused on the associations of catalase polymorphisms with various types of cancer, including cervical and prostate cancers. However, the results were inconsistent. To obtain a more reliable conclusion, we evaluated the relationship between the two common catalase gene polymorphisms (rs1001179 and rs794316) and cancer risk by a meta-analysis. Our meta-analysis included 37 published studies involving 14,942 cancer patients and 43,285 cancer-free controls. Odds ratios (ORs) and 95% confidence intervals (CIs) were used to evaluate the cancer risk. The results demonstrated that the rs1001179 polymorphism was associated with an increased cancer risk in the recessive and homozygote models (TT *vs*. CC: OR = 1.19, *P* = 0.01; TT *vs*. CT+CC: OR = 1.19, *P* <0.001). Furthermore, stratified analyses revealed a significant association between the rs1001179 polymorphism and prostate cancer in all models except the homozygote comparison. An association of the rs794316 polymorphism with cancer risk was detected in two genetic models (TT *vs*. AA: OR = 1.34, 95% CI = 1.03–1.74, *P* <0.001; TT *vs*. AT+AA: OR = 1.39, 95% CI = 1.09–1.77, *P* = 0.01). Additional well-designed studies with large samples should be performed to validate our results.

## INTRODUCTION

Worldwide, cancer is currently the main cause of death and a public health problem that seriously threatens human health [1]. Biological and epidemiological studies have shown that carcinogenesis is a sophisticated, multivariate process resulting from interactions between genetic and environmental factors [2]. However, the exact mechanism of carcinogenesis has not been fully elucidated. Many aspects of malignant cancers, including carcinogenesis, aberrant growth, metastasis, and angiogenesis, have been attributed to reactive oxygen species (ROS) [3]. Such ROS-mediated damage to cellular macromolecules is thought to accumulate as a function of age, thus promoting carcinogenesis [4, 5].

Catalase (CAT) is an important endogenous antioxidant enzyme that decomposes hydrogen peroxide to oxygen and water, thus limiting the deleterious effects of ROS[6]; accordingly, the *CAT* gene may play an important role in substance metabolism. *CAT* is located on the nuclear chromosome 11p13, and polymorphisms in this gene have been reported to associate with the development of many types of cancer, such as invasive cervical cancer and prostate cancer [7].

The rs1001179 polymorphism (C-262T) is located in the promoter region of *CAT*, where it influences transcription factor binding and alters the basal transcription and consequent expression of the encoded enzyme [8]. The rs794316 polymorphism (A-15 T) has been identified in the promoter region near the *CAT* start site, and the endogenous variability of this promoter likely plays a role in the host response to oxidative stress [9]. A large number of previous studies in humans have suggested a possible correlation between genetic polymorphisms of CAT and susceptibility to cancers, such as prostate cancer [10–14], breast cancer [15], and hepatocellular carcinoma [16–19]. However, those studies published inconsistent results. Accordingly, we conducted a meta-analysis to combine data from all of the available case-control studies in order to validate the association of *CAT* polymorphisms with cancer risk.

## RESULTS

### Characteristics of included studies

**Figure 1:**
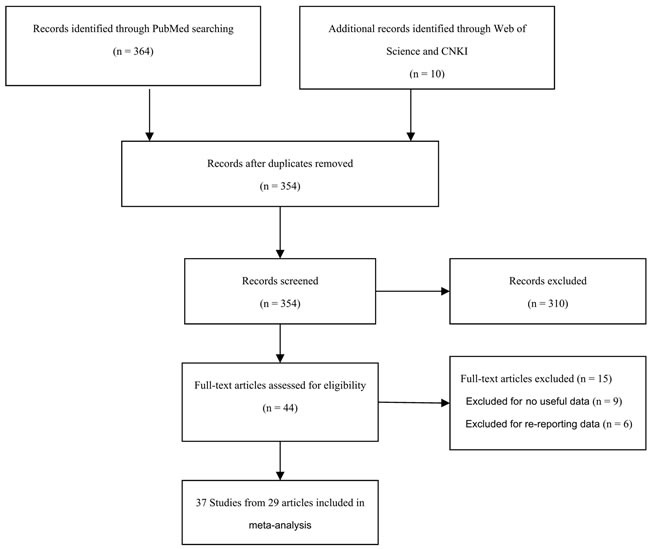
Flow diagram of included studies for the meta-analysis CNKI = China National Knowledge Infrastructure.

A flow chart of the study selection process is shown in Figure 1. Initially, 374 articles were identified. After reading the titles and abstracts of all the articles, 310 were excluded (164 articles were not related to cancer patients, 137 articles were not case-control studies and 9 articles were about other polymorphisms). After searching through the full texts of the remaining articles, an additional 15 were excluded, including 9 articles that contained no useful data and 6 articles that had re-reported data. Finally, a total of 37 studies from 29 published articles, involving 14,942 cases and 43,285 cancer-free controls, were included in this meta-analysis. The eligible studies presented data for several different cancer types, including prostate cancer, hepatocellular carcinoma, breast cancer, and colorectal cancer. Among these studies, 10 were based on Asian populations [9, 13, 15–17, 20–22], 20 on Caucasian populations [7, 10, 11, 14, 18, 23–33], 1 on an African population [14], and 6 on mixed-ethnicity populations [12, 19, 31, 34–36]. Furthermore, in 3 studies, the genotype distributions of the control groups departed from Hardy-Weinberg equilibrium (HWE) [7, 10, 20]. The characteristics of the eligible studies are presented in Table 1.

**Table 1:** Characteristics of the studies included in the meta-analysis

First author	Year	Country	Ethnicity	Genotyping medthod	Source of control	Cancer type	Total sample size (case/control)	HWE	SNP
Sousa	2016	Brazil	Mixed	Taqman	hospital	HCC	106/139	0.44	rs1001179
Castaldo	2015	Portugal	Caucasian	PCR	population	CC	119/106	**0.00**	rs1001179
Geybels	2015	Netherland	Caucasian	PCR	population	PC	1529/25184	**0.00**	rs1001179
Liu	2015	China	Asian	PCR-RFLP	hospital	HCC	266/248	0.68	rs1001179
Saadat	2015	Iran	Caucasian	PCR	population	BC	407/395	0.40	rs1001179
Su-1	2015	China	Asian	PCR-RFLP	hospital	HCC	301/186	0.49	rs1001179
Su-2	2015	China	Asian	PCR-RFLP	hospital	HCC	99/294	0.83	rs1001179
Banescu	2014	Romania	Caucasian	PCR-RFLP	population	CML	168/321	0.47	rs1001179
Aynali	2013	Turkey	Caucasian	PCR-RFLP	hospital	Laryngeal cancer	25/23	0.13	rs1001179
Tefik	2013	Turkey	Caucasian	PCR-RFLP	population	PC	155/195	0.07	rs1001179
Ding	2012	China	Asian	PCR	population	PC	1417/1008	0.86	rs1001179
Farawela	2012	Egypt	Caucasian	PCR-RFLP	population	NHL	100/100	0.49	rs1001179
Karunasinghe	2012	New Zealand	Mixed	Taqman	population	PC	258/567	0.42	rs1001179
Tsai	2012	Taiwan	Asian	PCR	hospital	BC	260/224	0.44	rs1001179
Chang	2012	China	Asian	PCR-RFLP	population	CRC	880/848	**0.00**	rs794316
Nahon	2011	France	Caucasian	Taqman	hospital	HCC	84/55	0.62	rs1001179
Ezzikouri	2010	France	Mixed	PCR-RFLP	population	HCC	96/222	0.59	rs1001179
He-1	2010	USA	Caucasian	Taqman	population	BCC	270/796	0.89	rs1001179
He-2	2010	USA	Caucasian	Taqman	population	Melanoma	211/796	0.89	rs1001179
He-3	2010	USA	Caucasian	Taqman	population	SCC	266/796	0.89	rs1001179
Tang	2010	USA	Mixed	Taqman	population	Pancreatic cancer	551/602	0.97	rs1001179
Wu	2010	Taiwan	Asian	PCR-RFLP	hospital	OCC	122/122	0.18	rs794316
Funke	2009	Germany	Caucasian	PCR	population	CRC	632/605	0.11	rs1001179
Li	2009	USA	Caucasian	Taqman	population	BC	497/493	1.00	rs1001179
Quick-1	2008	USA	Caucasian	HM L/I MS	population	BC	569/974	0.70	rs1001179
Quick-2	2008	USA	Mixed	HM L/I MS	population	BC	47/108	0.22	rs1001179
Rajaraman-1	2008	USA	Caucasian	Taqman	hospital	Glioma	330/438	0.57	rs1001179
Rajaraman-2	2008	USA	Caucasian	Taqman	hospital	Meningioma	120/438	0.57	rs1001179
Rajaraman-3	2008	USA	Caucasian	Taqman	hospital	Acoustic neuroma	63/438	0.57	rs1001179
Choi-1	2007	USA	Caucasian	HM L/I MS	population	PC	463/1233	0.26	rs1001179
Choi-2	2007	USA	African	HM L/I MS	population	PC	27/120	0.60	rs1001179
Cebrian	2006	UK	Caucasian	Taqman	population	BC	2171/2262	0.96	rs1001179
Ho	2006	China	Asian	PCR-RFLP	hospital	LC	230/240	0.44	rs1001179
Lightfoot	2006	USA/UK	Mixed	Taqman	population	NHL	909/1437	0.96	rs1001179
Ahn	2005	USA	Caucasian	HM L/I MS	population	BC	1008/1056	0.93	rs1001179
Lee-1	2002	South Korea	Asian	PCR-RFLP	population	GC	80/108	0.47	rs794316
Lee-2	2002	South Korea	Asian	PCR-RFLP	population	HCC	106/108	0.47	rs794316

PCR: polymerase chain reaction; RFLP: restriction fragment length polymorphism; HM L/I MS: high-throughput, matrixassisted, laser desorption/ionization time-of-flight mass spectrometry; HCC: hepatocellular carcinoma; CC: cervical cancer; BC: breast cancer; CML: chronic myeloid leukemia; NHL: non-Hodgkin lymphoma; BCC: basal cell carcinoma; SCC: squamous cell carcinoma; PC: Prostate cancer; CRC: colorectal cancer; OCC: Oral cavity cancer; GC: gastric cancer; LC: lung cancer; SNP: single-nucleotide polymorphisms; HWE: Hardy-Weinberg equilibrium.

### Meta-analysis of *CAT* polymorphisms and cancer risk

As shown in Table 2, the minor allele frequencies varied widely among cancer patients across the eligible studies, ranging from 0.04 to 0.50 for rs1001179 polymorphism and 0.31 to 0.43 for rs794316 polymorphism. The average minor allele frequencies for these polymorphisms were 0.19 and 0.40, respectively.

**Table 2:** Genotype Distribution and Allele Frequency of CAT polymorphisms in Cases and Controls

First author	Genotype (N)								Allele frequency (N)				MAF	
	Case				Control				Case		Control			
	total	AA	AB	BB	total	AA	AB	BB	A	B	A	B		
rs1001179														
Sousa 2016	106	68	35	3	139	103	32	4	171	41	238	40		0.19
Castaldo 2015	119	58	25	36	106	65	27	14	141	97	157	55		0.41
Geybels 2015	1529	887	539	103	25184	15794	8108	1282	2313	745	39696	10672		0.24
Liu 2015	266	239	27	0	248	223	24	1	505	27	470	26		0.05
Saadat 2015	407	261	129	17	395	240	132	23	651	163	612	178		0.20
Su-1 2015	301	273	27	1	186	168	18	0	573	29	354	18		0.05
Su-2 2015	99	92	7	0	294	264	29	1	191	7	557	31		0.04
Banescu 2014	168	105	49	14	321	168	132	21	259	77	468	174		0.23
Aynali 2013	25	13	10	2	23	12	11	0	36	14	35	11		0.28
Tefik 2013	155	58	64	33	195	107	68	20	180	130	282	108		0.42
Ding 2012	1417	1316	99	2	1008	940	67	1	2731	103	1947	69		0.04
Farawela 2012	100	26	49	25	100	28	53	19	101	99	109	91		0.50
Karunasinghe 2012	258	144	99	15	567	350	195	22	387	129	895	239		0.25
Tsai 2012	260	225	35	0	224	202	22	0	485	35	426	22		0.07
Nahon 2011	84	62	21	1	55	32	19	4	145	23	83	27		0.14
Ezzikouri 2010	96	76	14	6	222	173	45	4	166	26	391	53		0.14
He-1 2010	270	161	97	12	796	512	252	32	419	121	1276	316		0.22
He-2 2010	211	129	75	7	796	512	252	32	333	89	1276	316		0.21
He-3 2010	266	160	96	10	796	512	252	32	416	116	1276	316		0.22
Tang 2010	551	349	174	28	602	366	207	29	872	230	939	265		0.21
Funke 2009	632	374	235	23	605	348	231	26	983	281	927	283		0.22
Li 2009	497	295	176	26	493	303	167	23	766	228	773	213		0.23
Quick-1 2008	569	345	197	27	974	598	333	43	887	251	1529	419		0.22
Quick-2 2008	47	34	13	0	108	97	10	1	81	13	204	12		0.14
Rajaraman-1 2008	330	195	124	11	438	251	164	23	514	146	666	210		0.22
Rajaraman-2 2008	120	73	39	8	438	251	164	23	185	55	666	210		0.23
Rajaraman-3 2008	63	43	17	3	438	251	164	23	103	23	666	210		0.18
Choi-1 2007	463	281	157	25	1233	732	445	56	719	207	1909	557		0.22
Choi-2 2007	27	24	3	0	120	109	11	0	51	3	229	11		0.06
Cebrian 2006	2171	1351	707	113	2262	1362	787	113	3409	933	3511	1013		0.21
Ho 2006	230	209	19	2	240	217	23	0	437	23	457	23		0.05
Lightfoot 2006	909	554	298	57	1437	867	498	72	1406	412	2232	642		0.23
Ahn 2005	1008	614	349	45	1056	679	335	42	1577	439	1693	419		0.22
rs794316														
Chang 2012	880	280	448	152	848	272	472	104	1008	752	1016	680		0.43
Wu 2010	122	57	55	10	122	62	54	6	169	75	178	66		0.31
Lee-1 2002	80	35	38	7	108	51	44	13	108	52	146	70		0.33
Lee-2 2002	106	51	42	13	108	51	44	13	144	68	146	70		0.32

A: the major allele; B: the minor allele; MAF: minor allele frequencies.

**Figure 2:**
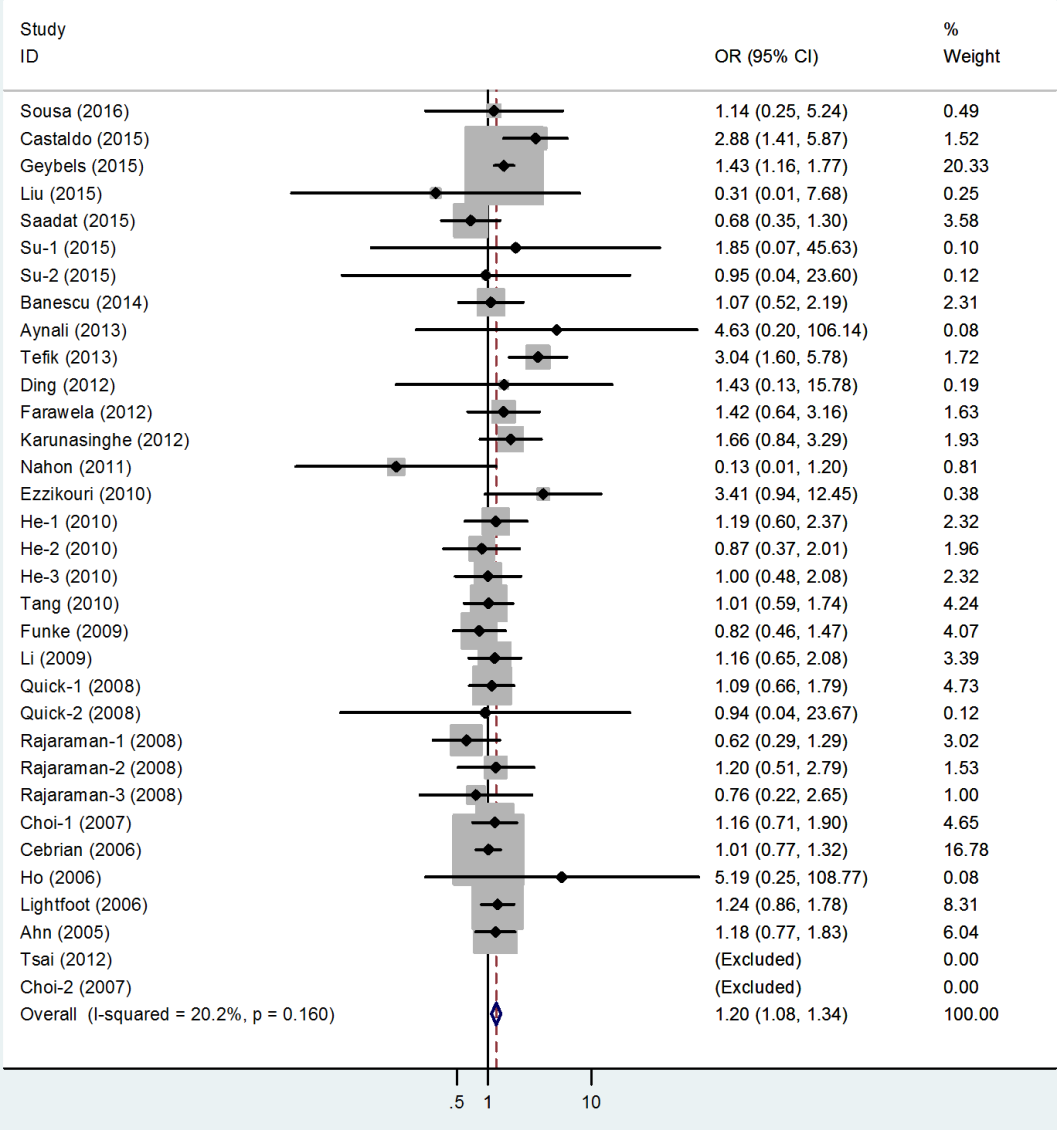
Forest plot of cancer risk related to rs1001179 polymorphism under TT versus CC genetic model T = the minor allele in rs1001179 polymorphism, C = the major allele in rs1001179 polymorphism, CI = confidence interval, OR = odds ratio.

The main results of this meta-analysis are listed in Table 3. Thirty-three studies involving 13,754 cases and 42,099 controls were included for rs1001179. As shown in Table 3 and Figure 2, we observed an increased cancer risk associated with the rs1001179 polymorphism under the homozygote and recessive models (TT *vs*. CC: odds ratio [OR] = 1.19, 95% confidence interval [CI] = 1.04-1.37, *P* = 0.01; TT *vs*. CT+CC: OR = 1.19, 95% CI = 1.06- 1.34, *P* < 0.001.) In the cancer-specific analysis, the results showed significant correlations between the rs1001179 polymorphism and prostate cancer risk in different comparison models (T *vs*. C: OR = 1.21, 95% CI = 1.04-1.41, *P* = 0.02; TT *vs*. CC: OR = 1.57, 95% CI = 1.17-2.10, *P* = 0.00; TT+CT *vs*. CC: OR = 1.20, 95% CI = 1.01-1.42, *P* = 0.04; TT *vs*. CT+CC: OR = 1.40, 95% CI = 1.18-1.67, *P* < 0.001). However, no meaningful correlations were observed in analyses stratified by ethnicity or the source of controls.

**Table 3:** Meta-analysis of the association between CAT polymorphisms and cancer risk

Comparisons	OR	95%CI	*P* value	Heterogeneity		Effects model
				I^2^	*P* value	
**B *vs* A**						
rs1001179	1.06	0.99-1.13	0.11	54%	0.00	R
HWE	1.04	0.97-1.11	0.28	39%	0.02	R
Caucasian	1.05	0.96-1.14	0.27	66%	0.00	R
Asian	1.05	0.86-1.29	0.64	0%	0.80	F
Mixed	1.10	0.92-1.32	0.29	54%	0.07	R
PC	**1.21**	**1.04-1.41**	0.02	61%	0.02	R
HCC	0.85	0.62-1.17	0.32	25%	0.25	F
BC	1.04	0.93-1.17	0.50	52%	0.05	R
rs794316	1.10	0.98-1.24	0.11	0%	0.88	F
HWE	1.06	0.84- 1.35	0.61	0%	0.76	F
**BB *vs* AA**						
rs1001179	**1.20**	**1.08-1.34**	0.00	20%	0.16	F
HWE	**1.12**	**1.00-1.27**	0.05	0%	0.70	F
Caucasian	1.16	0.97-1.38	0.10	41%	0.03	R
Asian	1.37	0.37-5.14	0.64	0%	0.80	F
Mixed	1.29	0.98-1.68	0.07	0%	0.47	F
PC	**1.57**	**1.17- 2.10**	0.00	33%	0.20	F
HCC	0.88	0.20- 3.82	0.87	45%	0.12	F
BC	1.03	0.85- 1.25	0.75	0%	0.82	F
rs794316	**1.34**	**1.03-1.74**	0.00	0%	0.58	F
HWE	1.09	0.62-1.91	0.76	0%	0.52	F
**AB *vs* AA**						
rs1001179	1.02	0.94- 1.09	0.68	39%	0.01	R
HWE	1.01	0.93- 1.09	0.82	35%	0.03	R
Caucasian	1.01	0.93- 1.11	0.76	47%	0.01	R
Asian	1.03	0.84- 1.28	0.77	0%	0.77	F
Mixed	1.05	0.80- 1.38	0.72	67%	0.02	R
PC	1.14	0.99- 1.31	0.06	33%	0.19	F
HCC	0.81	0.60- 1.09	0.17	0%	0.73	F
BC	1.07	0.91- 1.25	0.43	60%	0.02	R
rs794316	0.97	0.81- 1.16	0.74	0%	0.76	F
HWE	1.10	0.79- 1.52	0.59	0%	0.81	F
**BB+AB *vs* AA**						
rs1001179	1.04	0.96- 1.12	0.33	48%	0.00	R
HWE	1.02	0.95- 1.11	0.54	39%	0.02	R
Caucasian	1.03	0.94- 1.14	0.50	59%	0.00	R
Asian	1.04	0.84- 1.29	0.70	0 %	0.79	F
Mixed	1.09	0.86- 1.38	0.49	62%	0.03	R
PC	**1.20**	**1.01- 1.42**	0.04	55%	0.05	R
HCC	0.83	0.62- 1.11	0.21	0%	0.56	F
BC	1.06	0.91- 1.23	0.44	59%	0.02	R
rs794316	1.04	0.87-1.23	0.68	0%	0.92	F
HWE	1.10	0.80- 1.49	0.57	0%	0.85	F
**BB *vs* AB+AA**						
rs1001179	**1.19**	**1.06- 1.34**	0.00	10%	0.31	F
HWE	**1.12**	**1.00- 1.27**	0.05	0%	0.70	F
Caucasian	1.16	0.99- 1.35	0.06	29%	0.11	F
Asian	1.38	0.37- 5.18	0.63	0 %	0.80	F
Mixed	1.30	0.99- 1.70	0.05	0%	0.50	F
PC	**1.40**	**1.18- 1.67**	0.00	0%	0.48	F
HCC	0.95	0.23- 3.99	0.94	43%	0.14	F
BC	1.04	0.86- 1.25	0.70	0%	0.89	F
rs794316	**1.39**	**1.09-1.77**	0.01	0%	0.41	F
HWE	1.05	0.61- 1.79	0.87	0%	0.46	F

A: the major allele; B: the minor allele; F: fixed effects mode; R: random effects model; HCC: hepatocellular carcinoma; BC: breast cancer; PC: Prostate cancer; HWE: meta-analysis excluding the studies departing from HWE.

**Figure 3:**
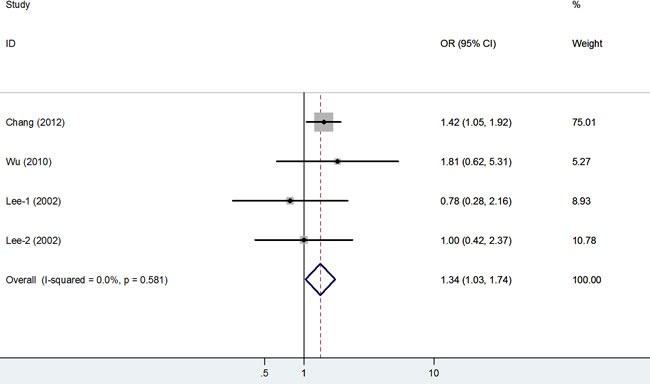
Forest plot of cancer risk related to rs794316 polymorphism under TT versus AA genetic model T = the minor allele in rs794316 polymorphism, A = the major allele in rs794316 polymorphism, CI = confidence interval, OR = odds ratio.

The association of the rs794316 polymorphism with cancer risk was investigated in 4 studies involving 1,188 cases and 1,186 controls. This polymorphism was associated with an increased cancer risk in the overall population under the two models (TT *vs*. AA: OR = 1.34, 95% CI = 1.03-1.74, *P* < 0.001; TT *vs*. AT+AA: OR = 1.39, 95% CI = 1.09-1.77, *P* = 0.01; Figure 3).

### Heterogeneity analysis and publication bias

**Figure 4:**
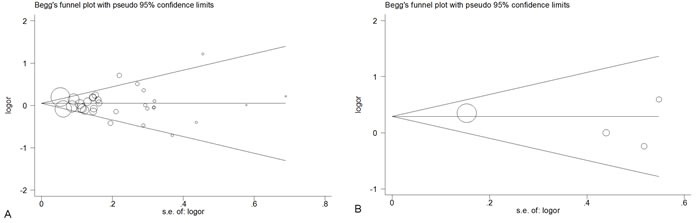
Begg's funnel plot for publication bias test of CAT polymorphisms: rs1001179 (A), rs794316 (B), under the homozygous model

In this meta-analysis, Q-statistic test was used to detect between-study heterogeneity that arose from methodological or clinical dissimilarity across studies. When the P value of the heterogeneity test was more than 0.1 (P ≥0.1), a fixed-effects model was performed. Otherwise, the random-effects model was used. To explore the other factors which may influence our results, we performed a meta-regression analysis. As shown in the Table 4, sample size was not the factor which could be involved in cancer susceptibility (*P* = 0.134). Furthermore, the results revealed that the publication year, ethnicity, genotype method and the source of controls were all not the factors that could impact on our results (*P* = 0.088, 0.368, 0.676 and 0.300, respectively). We also performed a funnel plot and Egger's test to assess publication bias. As shown in Figure 4, the funnel plots failed to reveal any obvious asymmetries of the 2 polymorphisms in the overall population, and the results of Egger's test revealed no publication bias (*P* > 0.05). Therefore, the results revealed that publication bias was not significant in this meta-analysis.

**Table 4:** Meta-regression analyses of potential source of heterogeneity

Heterogeneity factors	Coefficient	SE	Z	*P*	95% CI	
					LL	UL
Sample size	0.047	0.042	1.12	0.273	−0.039	0.134
Publication year	0.026	0.014	1.77	0.088	−0.004	0.056
Ethnicity	0.146	0.159	0.92	0.368	−0.182	0.473
Genotype method	−0.023	0.054	−0.42	0.676	−0.135	0.089
Source of control	0.259	0.244	1.06	0.300	−0.244	0.761

SE: standard error; 95% CI: 95% confidence interval; LL: lower limit; UL: upper limit.

### Sensitivity analysis

**Figure 5:**
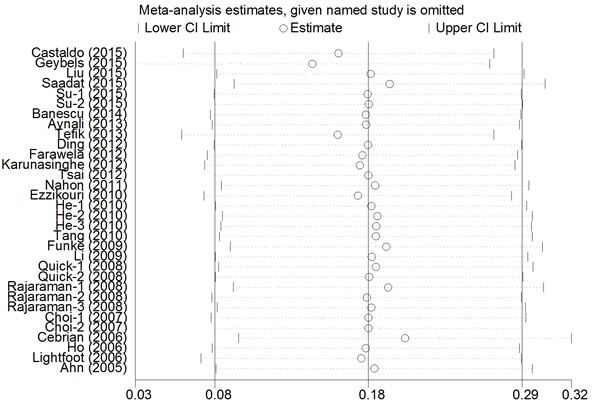
Sensitivity analysis of the association between CAT rs1001179 polymorphism and cancer risk under the homozygous model

A single study was deleted one at a time from the meta-analysis to reflect the influence of each individual dataset on the pooled ORs. The analysis results demonstrated that no single study greatly influenced the overall cancer risk estimations with respect to the *CAT* polymorphisms (Figure 5), which indicates that our results are statistically robust.

## DISCUSSION

Previous case-control studies have investigated the association between the rs1001179 polymorphism and cancer risk. No significant associations were observed between rs1001179 polymorphism and hepatocellular carcinoma or breast cancer risk in studies by Liu et al. [17] and Saadat et al. [23], respectively. However, Geybels et al. [10] and Castaldo et al.[7] reported significant associations between rs1001179 polymorphism and increased prostate and cervical cancer risks, respectively, and Nahon et al. [18] and Su et al. [16] demonstrated that rs1001179 polymorphism was a protective factor with respect to hepatocellular carcinoma susceptibility.

We combined all the case-control studies concerning rs1001179 polymorphism and cancer risk to perform this meta-analysis, and found that individuals harboring the rs1001179 TT and rs794316 TT genotypes had a higher cancer risk than did those with other genotypes. This is likely attributable to the relationship between rs1001179 polymorphism and lower *CAT* activity, which further hinders the response to oxidative stress and might lead to tumorigenesis [37, 38]. The stratified analysis results indicated that the *CAT* rs1001179 polymorphism was only associated with prostate cancer, but not other cancers. These results were in accordance with others' findings. Geybels et al. observed that the *CAT* rs1001179 polymorphism was associated with the risk of stage III/IV prostate cancer, which might be explained by the effect of *CAT* expression on oxidative stress and the link between increased oxidative stress and prostate cancer.

A previous meta-analysis including 9,777 cancer patients and 12,223 controls showed significant association between rs1001179 polymorphism and cancer risk in the recessive model [39]. Compared with that meta-analysis, our meta-analysis included 11 new independent studies of hepatocellular carcinoma [16, 17, 22, 34], chronic myeloid leukemia [24], laryngeal cancer [25], colorectal cancer [20], and oral cavity cancer [9]. Different from the previous result, we observed an association between the rs1001179 polymorphism and an increased cancer risk in the homozygote model. And it is worth mentioning that we found an association of the rs794316 polymorphism with cancer risk in recessive model and homozygote model, which wasn't detected by anyone before.

Because the control group genotype distributions departed from HWE in 3 studies, we performed a subgroup analysis that excluded those studies. Regarding the rs1001179 polymorphism, the result was remained consistent with the overall analysis; in other words, an association between an increased cancer risk and rs1001179 polymorphism was observed in recessive model and homozygote model. Nevertheless, we observed no significant association between the rs794316 polymorphism and cancer risk with any of the genetic models, although this might be a consequence of the small number of studies.

Several limitations of this meta-analysis should be acknowledged. First, only Asian population was involved in the analysis of rs794316, and most studies of rs1001179 are for Caucasian and Asian population. Accordingly, it would be better to include more studies with various ethnic groups to identify their definite roles in different populations. Second, some detailed information (e.g., sex, age, lifestyle, and environmental factors) was not considered. Third, the overall outcomes were based on individual unadjusted ORs, whereas a more precise evaluation should be adjusted using other potentially suspect factors. Fourth, the genotyping methods used in the eligible studies differed widely, which might have influenced the results. Moreover, although we have summarized all data on rs794316 polymorphism and cancer risk, the number of relative studies still needs further expansion.

In summary, this meta-analysis has shown associations of the *CAT* rs1001179 and rs794316 polymorphisms with an increased cancer risk. Additional larger-scale multicenter studies with larger sample sizes are needed to further validate the possible roles of these polymorphisms in cancers.

## MATERIALS AND METHODS

### Search strategy

The PubMed, Web of Science, and Chinese National Knowledge Infrastructure (CNKI) databases were searched for publications from 2002 to January 2016 using the terms “cancer” or “tumor”, “CAT” or “Catalase”, “polymorphism” or “SNP”, “rs1001179” or “C-262T”, and “rs794316” or “A-15 T”. We also used the “Related Articles” option in PubMed to identify additional studies of the same topic. The reference lists of the retrieved articles were also screened. All included studies were selected using the following criteria: (a) studies must have featured a case-control design and focused on *CAT* polymorphism and cancer risk; (b) published data must have been sufficient to allow OR estimation with a 95% CI; and (c) for multiple publications reporting the same data or overlapping data, the largest or most recent publication was selected.

### Data extraction

Initially, 2 investigators (Liu K and Liu XH) independently checked all potentially relevant studies, and disagreements were resolved through discussions with a third researcher. We extracted the following items from each article: first author, year of publication, country of origin, ethnicity, cancer types, control source, genotyping method, total numbers of cases and controls, and numbers of different genotypes among cases and controls. All data were extracted from published articles. All cancers were confirmed by histology or pathology. The non-cancer controls had no evidence of any malignant disease at the time of the study.

### Statistical analysis

We used ORs and 95% CIs to evaluate the cancer risks associated with *CAT* polymorphisms. Heterogeneity between studies was evaluated using the I^2^ test, with a higher I^2^ value indicating a higher level of heterogeneity (I^2^ = 75-100%: extreme heterogeneity; I^2^ = 50-75%: great heterogeneity; I^2^ = 25-50%: moderate heterogeneity; I^2^ < 25%: no heterogeneity). During the heterogeneity evaluation, the fixed-effects model would be used if the P value was ≥0.10; otherwise, the random-effects model was used. Subgroup analyses were performed according to cancer type, control source, and ethnicity. A sensitivity analysis was performed to assess the stability of the final results by sequentially omitting each individual study at a time. Egger's test and Begg's test were adopted to assess publication bias. The meta-analysis assessed the following genetic models: dominant model (AB+BB *vs*. AA), recessive model (BB *vs*. AA + AB), homozygote comparison (BB *vs*. AA), heterozygote comparison (AB *vs*. AA), and allele comparison (B *vs*. A). All analyses were performed using the Stata software, version 12.0 (Stata Corp., College Station, TX, USA). A P value < 0.5 was considered statistically significant, and all P values were 2-sided.
